# Salivary Assessments in Post-Liver Transplantation Patients

**DOI:** 10.3390/jcm11113152

**Published:** 2022-06-01

**Authors:** Andreea Cristiana Didilescu, Adelina Lazu, Cristian Vlădan, Cristian Scheau, Laura Dan Popa, Petra Șurlin, Wendy Esmeralda Kaman, Hendrik Simon Brand

**Affiliations:** 1 Department of Embryology, Faculty of Dental Medicine, Carol Davila University of Medicine and Pharmacy, 8 Eroii Sanitari Boulevard, 050474 Bucharest, Romania; adelina_lazu@yahoo.com; 2 Department of Oral and Maxillofacial Surgery, Dan Theodorescu University Hospital of Oral and Maxillofacial Surgery, 19 Calea Plevnei Street, 010221 Bucharest, Romania; 3 Department of Physiology, Carol Davila University of Medicine and Pharmacy, 8 Eroii Sanitari Boulevard, 050474 Bucharest, Romania; cristian.scheau@umfcd.ro; 4 Department of Liver Transplant, St. Mary’s Clinical Hospital, 37–39 Ion Mihalache Boulevard, 011172 Bucharest, Romania; ldanpopa@yahoo.com; 5 Department of Periodontology, Faculty of Dental Medicine, University of Medicine and Pharmacy of Craiova, 2 Petru Rareș Street, 200349 Craiova, Romania; surlinpetra@gmail.com; 6 Department of Oral Biochemistry, Academic Centre for Dentistry Amsterdam (ACTA), VU University of Amsterdam and University of Amsterdam, Gustav Mahlerlaan 3004, 1081 LA Amsterdam, The Netherlands; w.e.kaman@acta.nl (W.E.K.); h.brand@acta.nl (H.S.B.)

**Keywords:** saliva, liver transplant, oral health, questionnaire

## Abstract

Saliva is in the first line of the body’s defense mechanism. In order to better understand how liver transplantation impacts salivary biochemistry, the aim of this cross-sectional study was to explore variations of salivary markers for oral health in post-liver transplantation patients, as compared with systemically healthy dental outpatients (controls). In this case, 26 patients were enrolled in each group, with similar socio-demographic characteristics. Unstimulated whole saliva was collected; total protease activity and total protein content were measured. The oral health in both groups was assessed using a self-report oral health questionnaire. Data were analyzed using parametric and nonparametric tests. Comparable results were recorded in terms of salivary protein and protease activity assessments. In post-liver transplantation group, positive correlation was found between the salivary pH level and the salivary secretion rate (*r* = 0.39; *p* = 0.04). With respect to self-reported oral health, there were no significant differences between the two groups, except for dental and oral care habits, the controls reporting more frequently use of dental floss and mouthwash (*p* = 0.02, and *p* = 0.003, respectively). Considering the high risk for developing systemic complications after liver transplantation, oral health care is an important issue to be addressed, salivary investigations representing powerful tool for disease changes monitoring.

## 1. Introduction

Saliva is in the first line of the body’s defense mechanism. The salivary glands secrete many proteins with antimicrobial activity, as well as peptides with potential of stimulating the fibroblasts and the epithelial cells, thereby enhancing wound healing [1,2]. In addition, saliva contains proteins derived from plasma, which means that saliva is an attractive alternative fluid for the determination of some plasma markers’ concentrations which are currently used for diagnostic purposes. Saliva can be collected patient-friendly, rapidly, frequently and non-invasively, and no medically trained personal is needed for collection. However, the unstimulated salivary secretion sampling protocol still requires specific indications for patients in order to preserve the integrity of the biological samples [3]. Together, this indicates that salivary screening tests have the potential to detect and provide an indicative diagnosis in very short periods of time [4]. The potential of saliva for use as a diagnostic tool for systemic diseases has been recently reinforced in a study conducted on liver-transplanted children and healthy controls. Inflammatory burden markers were analyzed in blood and salivary samples; the findings revealed similar pattern of the salivary inflammatory markers to the serum inflammatory values [5].

Patients receiving a liver transplant require long-term immunosuppressive drug therapy to prevent immune rejection of the transplant. In addition to the effects of liver transplantation itself, the post-liver transplant-associated immunosuppressive medication and the administered antihypertensive therapy, in combination with comorbidities, may induce a reduction of the salivary secretion rate [6]. Stressful situations, such as dental examinations, may also negatively influence the salivary secretion rate [7]. The decreased salivary secretion negatively influences the state of oral health and can predispose patients to increased incidence of periodontal disease, dental caries, the occurrence of alterations such as dysphagia and oral discomfort, and fungal superinfections [4,8].

Patients with liver transplantation exhibit important effects with respect to oral health, such as increased gingival inflammation and increased risk for oral cancer occurrence, when compared to healthy patients [9,10]. From a biological point of view, severe periodontitis seems to be the type of periodontal disease in close relationship with general health [11]. Due to prolonged hospitalization and poor general condition, clinical periodontal examination is difficult to be performed among patients undergoing liver transplantation. In this situation, patient self-reported information may be considered useful tool for periodontal disease surveillance [12,13]. Thus, a questionnaire consisting of 8 closed-ended items has been proposed [14], and validated for patients in a medical care setting [11]. However, other studies stated that self-reports appear to be less useful for the assessment of periodontal disease and may vary among populations [15,16]. The performance of these questionnaires also seems better with respect to predicting severe periodontitis compared with moderate disease. Therefore, there is still need for an objective non-invasive screening of the condition of the periodontium [17]. Periodic analysis of saliva would be an option to monitor the condition of the periodontium, as it has been suggested that total protein concentration and salivary protease activity are related to the severity of inflammation of the periodontium [18,19].

In order to better understand how liver transplantation impacts salivary biochemistry, the aim of the study was to explore variations of salivary markers for oral health in post-liver transplantation patients, as compared with systemically healthy dental outpatients.

## 2. Materials and Methods

### 2.1. Study Design

The present study was conducted between 1 November 2019 and 15 March 2020. Study design was cross-sectional. Two patient groups were recruited: post-liver transplantation patients (LTx, study group) selected from the Department of Liver Transplant, St Mary’s Clinical Hospital, Bucharest, and systemically healthy patients (control group) referred to a dental private clinic from Bucharest. A priori sample size calculation was performed using G*Power software, version 3.1.9.4 (Heinrich-Heine-Universität Düsseldorf, Düsseldorf, Germany); with an effect size of 0.75 and a power of 80%, 24 participants were needed in both experimental groups. Each subject received information regarding the development of the study, and written and verbal consent were obtained. In both groups, patients aged between 18 and 70 years old were included in the study. For the LTx patients, exclusion criteria comprised central nervous system disorders and/or severe alterations of general health status and the impossibility of performing oral procedures. The study was conducted in accordance with the Declaration of Helsinki, and the protocol was approved by the Institutional Ethics Committee of Carol Davila University of Medicine and Pharmacy (no. 227/21/10/2019).

A single trained examiner (A.L.) performed all the procedures. For the subjects in the LTx group, the day of saliva collection coincided with the time of recurrent admission for check-ups, and all procedures were performed in the hospital. Similarly, saliva samples from control patients were collected in the dental office, prior examination and application of questionnaires.

Postprandial salivary collection was performed at least one hour after the meal, within a time of 5 min. Similar to the protocol for saliva collection described in the specialty literature, the patients were encouraged to keep their mobile or mobilizable dentures on [7]. A rigorous protocol was developed and applied according to other studies in the field, to prevent a potential distortion of the obtained results [20]. Olfactory stimuli, the exaggerated water consumption or the position of the body were factors evaluated prior to the establishment of the clinical protocol [21]. Moreover, the patients were instructed to avoid the consumption of food, water or juices, chewing gum or mouthwash before the collection of saliva [21].

The polypropylene containers used for collection of saliva samples were sterile, had a volume of 30 mL, were individually packaged and graded to quantify the individual volume of the samples by visual inspection. Each container was inscribed with the number of the corresponding subject included in the study for easy identification purposes. The containers were weighted before and after the salivary sample collections and the difference between the initial weight prior to the collection and the final weight was calculated; the value of the salivary secretion rate resulted by dividing the result by the number of minutes dedicated to the collection of saliva (5 min) [7,22]. After collection, the pH of the samples was determined by immersing universal indicator papers, with 0–14 pH range, into the salivary contents. Afterwards, the samples were stored at a constant temperature of −80 °C until the initiation of the laboratory analyses. For the analyses, the initial containers were slowly thawed at room temperature and transferred with calibrated pipettes into the sterile calibrated Eppendorf tubes with a volume of 2 mL and centrifuged at 3500× *g* for 15 min.

### 2.2. Total Protein Content

Assessment protocol for total protein content has been previously described [23]. Measurements were performed using the Pierce™ BCA Protein Assay Kit (Thermo Scientific, Landsmeer, The Netherlands) in 96-well polystyrene microplates (Greiner Bio-One, Alphen aan de Rijn, The Netherlands). Each saliva sample was initially 1:1 diluted in Phosphate Based Saline (PBS), and subsequently 20 µL of the diluted saliva samples was transferred to the wells of the microplate. Next, 180 µL of the BCA reagent was added to each well. A standard curve ranging from 0 to 1500 µg/mL was prepared by serial dilutions of bovine serum albumin (BSA, Merck, Amsterdam, The Netherlands) in PBS. After 30 min incubation at 37 °C, optical readouts at 405 nm were obtained using a microplate photometer (Multiskan™, Thermo Scientific, Landsmeer, The Netherlands). All saliva samples were analyzed in duplicate, and results were expressed in µg/mL.

### 2.3. Protease Analysis

Total salivary protease activity was performed based on the cleavage of fluorescence resonance energy transfer (FRET) substrates [24]. The protocol followed was previously described [23]. Briefly, 49 µL saliva was incubated with 1 µL of 800 µM PEK-054 ([FITC]-NleKKKKVLPIQLNAATDK-[KDbc]) or PFU-089 ([FITC]-FR-[KDbc]). The fluorescence of each well was read for 1 h at 37 °C on a fluorescence microplate reader (FLUOstar Galaxy, BMG Laboratories, Offenburg, Germany) with an excitation wavelength of 485 nm and an emission wavelength of 530 nm. Results were expressed as the increase in fluorescence per minute (F/min). All saliva samples were analyzed in duplicate.

### 2.4. Self-Reported Oral Health Questionnaire

The Romanian version of a self-reported questionnaire developed by Eke et al. [14], and adjusted by Verhulst et al. [11], was completed by all patients, as previously described [13].

### 2.5. Statistical Analyses

Statistical analyses were performed using Stata/IC 16 (StataCorp. 2019. Stata Statistical Software: Release 16. College Station, TX, USA: StataCorp LLC). Data distributions were expressed as means, standard deviations, intervals, medians and percentages. Quantitative variables were tested for normal distribution using the Shapiro-Wilk Test. Intergroup comparisons were carried out using unpaired Student *t*-test and Mann–Whitney *U* test. Correlations between variables were explored using Pearson (*r*) correlation coefficient. For categorical measures, Pearson Chi-squared test was used. Fisher exact test was used when the expected frequency of any cell in the table was <5. A *p*-value < 0.05 was considered statistically significant.

## 3. Results

### 3.1. Characteristics of the Study Groups

In this case, 26 LTx patients and 26 dental patients were included in this study. Patients’ background is shown in Table 1. There were no significant differences between the two groups in terms of age, gender, smoking, rural background, tertiary education, and frequency of dental check-ups per year (Student *t*-test, Pearson Chi-squared and Fisher exact tests, *p* > 0.05).

In the LTx group, the average time between the date of the transplant intervention and saliva collection was 38.4 months (±22.1; range 1–70). All LTx patients were chronic liver transplantation recipients and received Tacrolimus as immunosuppressive medication.

### 3.2. Salivary Assessments

**Figure 1:**
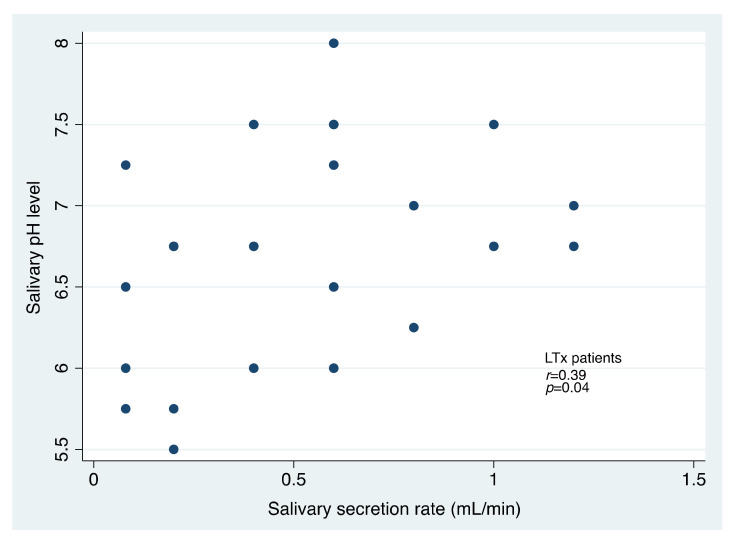
Scatter plot illustrating the relationship between salivary pH level and salivary secretion rate in patients receiving a liver transplant (LTx, n = 26).

The rate of unstimulated salivary secretion was similar in both groups (0.51 ± 0.35 mL/min in LTx patients, and 0.55 ± 0.33 mL/min in controls, respectively; *p* = 0.69, Student *t*-test). Although the mean salivary pH was higher in LTx patients than in the controls, the difference was not statistically significant (6.68 ± 0.67 versus 6.42 ± 0.57; *p* = 0.14, Student *t*-test). A statistically significant positive correlation was recorded between the salivary pH level and the salivary secretion rate in the LTx group (*r* = 0.39; *p* = 0.04, Pearson correlation coefficient) (Figure 1). A positive correlation was also found in the control group, without statistical significance (*r* = 0.27; *p* = 0.18, Pearson correlation coefficient).

**Figure 2:**
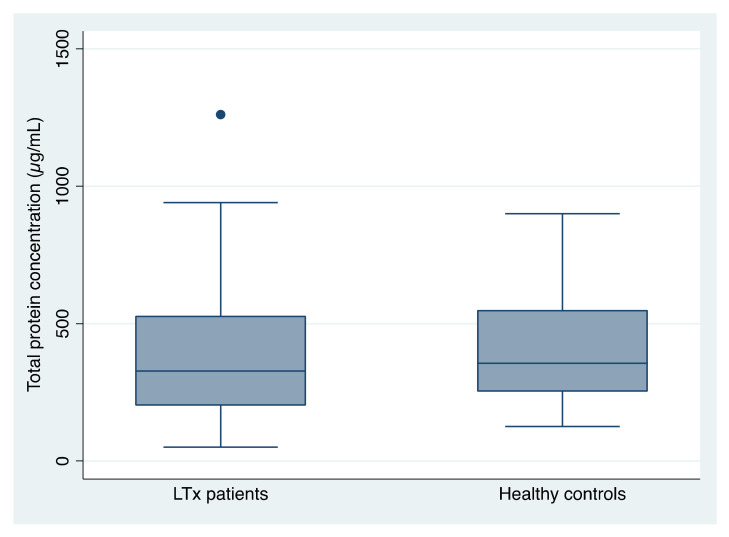
Total protein concentration in unstimulated whole saliva of patients receiving a liver transplant (LTx) and healthy controls (both n = 26).

The average salivary protein concentration in LTx patients was 414.03 µg/mL (±297.57; range 50–1261; median 328). The average salivary protein concentration in healthy controls was 397.65 µg/mL (±184.2; range 126–900; median 355.5) (Figure 2). There were no statistically significant differences between the two groups (*p* > 0.05; Mann-Whitney U test).

**Figure 3:**
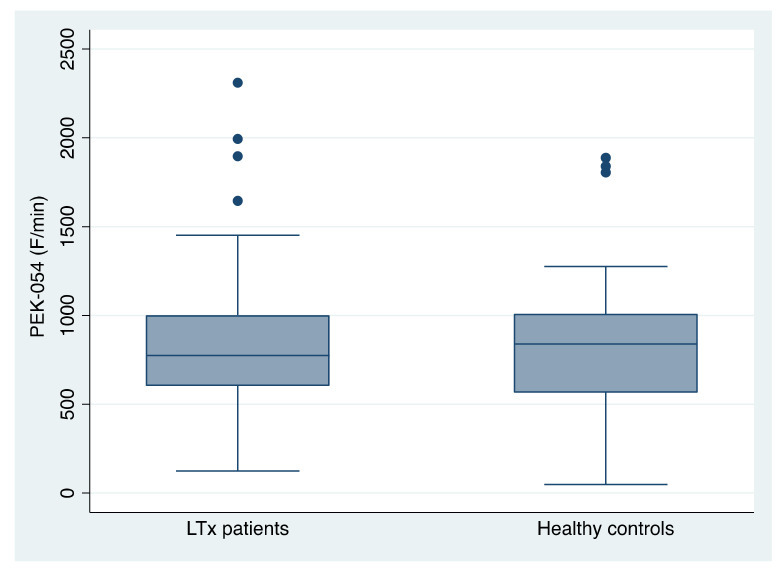
Salivary total protease activity using substrate PEK-054, in unstimulated whole saliva of patients receiving a liver transplant (LTx) and healthy controls (both n = 26).

The mean total proteolytic activity using PEK-054 was 903.58 F/min (±543.76; range 124.8–2310.3; median 773.7) in LTx patients versus 857.62 F/min (±471.79; range 48.5–1887.7; median 838.75) in healthy controls (Figure 3). There were no statistically significant differences between the groups (*p* > 0.05; Mann-Whitney U test).

**Figure 4:**
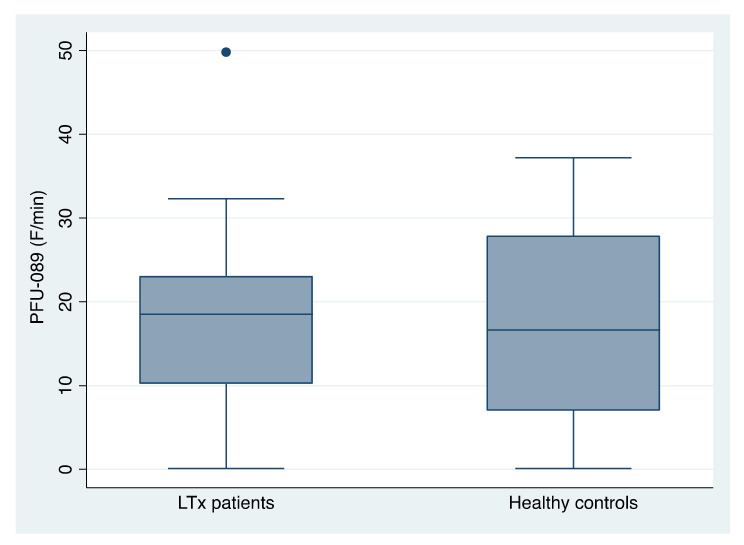
Salivary protease activity using substrate PFU-089 in unstimulated whole saliva of patients receiving a liver transplant (LTx) and healthy controls (both n = 26).

The mean proteolytic activity using the PFU-089 substrate in LTx patients was 18.09 F/min (±11.07; range 0.1–49.8; median 18.5) versus 17.91 F/min (±12.41; range 0.1–37.2; median 16.65) in healthy controls (Figure 4). There were no statistically significant differences between the groups (*p* > 0.05; Student *t*-test).

### 3.3. Self-Reported Oral Health (SROH)

The results for SROH questionnaire responses and intergroup comparisons are shown in Table 2. There were no significant differences observed between the two groups, except the dental and oral care habits.

## 4. Discussion

In terms of its benefits, saliva is a remarkable environment for the assessment of the liver function, especially because it has a rapid and non-invasive collection procedure. Despite this, for the test performed in the present study, it is important to mention a limitation corresponding to the data in the literature, namely that salivary proteolytic enzymes have the potential of influencing the values of the determined markers [4,25]. The qualitative or quantitative assessments were tests performed outside the oral cavity on unstimulated saliva samples. For this reason, the evaluation of the salivary pH had a series of notable limitations, such as: the pH reference value is general, without any reference to the differences between the intraoral and extraoral environment; the extraoral environment determines disturbances in the salivary buffer systems; there are variations of the salivary pH values at the level of the soft and hard structures of the oral cavity and the secreted saliva [26]. The use of electronic pH meters for the calculation of the salivary pH is an optimal method for obtaining conclusive results, but the data in the specialty studies reveal comparable values by determination by means of indicator strips [27]. In the present study, the mean value of the salivary pH obtained in the LTx group (6.68) was slightly increased compared to the mean value of the control group (6.42). This difference is consistent with the results of other studies and it has been attributed to the more abundant tartar deposits in LTx patients and associated immunosuppressive therapy [28].

The mean of salivary flow rate was slightly higher in controls than in LTx, without statistically significant difference. The reference values for the unstimulated salivary secretion rate range from 0.3 to 0.5 mL/min [29,30]. It was previously reported that patients with chronic liver failure may have a lower salivary flow [31], but studies on alcoholic cirrhosis revealed higher flow rates, most likely due alcohol-induced parotid hypertrophy [32]. Reduced unstimulated saliva was reported in a higher percentage of chronic liver transplantation recipients as compared with acute liver transplantation recipients [33]. In another study, salivary flow was found preserved for the majority of solid-organ transplant recipients, including LTx patients [34]. It is interesting that in the case of salivary secretion rate, simple tooth brushing entails an increase that normalizes after 15 min [35].

With regard to the positive association recorded between the pH and salivary flow rate in LTx patients, high values of these measurements, together with poor oral hygiene, could expose such individuals to higher risk for developing periodontitis [36].

### 4.1. The Total Protein Concentration

The central element for the functioning of saliva is its content in proteins that play a role in all the processes specific to this biological environment [37]. The systemic condition, either the healthy context or the presence of a disease influence, impacts the proteome to a small degree, with minor structural differences at the level of proteins. The current laboratory tests however have the advantage of a relevant potential to discriminate between these small amplitude protein differences [38].

The saliva collection protocol is an important detail for the integrity of the samples. A comparison between the different saliva collection methods reveals minor changes in the protein composition of the unstimulated salivary secretion [4].

In the present study, although the mean values recorded were slightly higher in the case of LTx patients than in the control patients, there were no statistically significant differences, as reported in a previous study performed on young recipients of liver transplants [28]. Based on the comparable salivary total proteins concentrations and salivary flow rates in LTx patients and controls, we suggest that the salivary glands function was comparable in both groups.

### 4.2. Protease Activity

Given the fact that L-amino acids form most of the amino acids identified in natural proteins, a limitation of the present study regarding the protease activity in saliva is that the peptide substrates used in this study can be hydrolyzed by both human and bacterial proteases [39]. Substrate PEK-054 is a long substrate (approximately 20 amino acids) which translates into the fact that proteases have several sites available to exert their cleavage effect. On the other hand, substrate PFU-089 is a shorter substrate (only 3 amino acids) so there are fewer selectively available recognition and binding sites for proteases. For this reason, the proteolytic activity determined with the substrate PFU-089 is much lower than the activity determined using the PEK-054 substrate [40,41,42]. The present study does not reveal significant differences between the two groups regarding the protease activity for either of the two substrates used for this purpose.

It has been suggested that the salivary protease activity is influenced by liver transplantation, as shown by Ziebolz et al. [43], which found significantly lower MMP-8 concentrations in post-liver transplantation patients compared to the pre-liver transplantation group in a sublot of patients with moderate periodontitis. However, it is unclear whether the liver transplantation or the associated immunosuppression is responsible for the variation of protease activity. In addition, the periodontal conditions seem to play a major role in the concentration of salivary proteases [44,45], therefore acting as a confounding variable when monitoring the dynamics in liver transplantation. In a study performed on 84 liver transplantation patients, MMP-8 levels were higher in patients with impaired periodontal health than in patients with good periodontal health, as well as the MMP-8/TIMP-1 molar ratios. Their values were correlated with stimulated salivary flow rates, which were the highest in stage II and III periodontitis. Salivary albumin and total protein concentrations, however, were not significantly different between healthy and affected periodontium cases in LTx patients [46]. Literature data regarding the correlation between salivary protein concentration and protease activity after liver transplantation is scarce. While liver transplantation is expected to normalize serum protein levels, there is an interference between salivary gland alterations in chronic liver failure, salivary flow and protein concentration variations, as well as periodontal disease that may cause unpredictable effects on the saliva composition in LTx patients. Further studies accounting for all these variables are required to clarify this issue.

### 4.3. Self-Reported Oral Health (SROH) in Relationship with Salivary Assessments

Oral and dental health may be affected by immunosuppression in LTx patients. Sepsis is an important cause of death in organ transplant patients, oral opportunistic infections being more frequently encountered in these patients [10,47]. It has been demonstrated that, after liver transplantation, patients have a five-fold increased risk for oral cancer occurrence compared to the general population [10]. To reduce the risk of systemic complications with an oral origin, maintenance of an optimal oral health is important for LTx patients [47,48,49] Loss of bone mass has been reported with end-stage liver disease and also during first months after liver transplantation. Nevertheless, in patients with normal graft function, bone metabolism improves, and bone loss decreases during the first year after transplant [50].

Our results from SROH questionnaire support salivary findings with respect to the similarities between the two groups. SROH has been chosen as a screening tool for periodontitis, previously designed for use by medical professionals in non-dental settings [11]. Due to the impossibility of a proper oral examination of LTx patients, this tool has been used in order to depict the oral health as well as possible. However, SROH estimates should be interpreted and compared with caution, considering regional and cultural particularities. In a previous pilot study, we showed that there were no significant differences between LTx patients and healthy controls according to their self-perception of oral health [13]. In another study performed in Finland, chronic LTx patients reported their oral health to be worse as compared with controls [8]. In the same study, worse oral health was associated with hyposalivation in chronic transplant recipients [8]. Still, deficiencies in oral hygiene behavior noted in our LTx patients require an increased attention to oral health care, fact already mentioned before [51].

Weaknesses of the study include the small sample sizes of the groups. Due to the COVID-19 pandemic, our recruitment possibilities were restricted. We should also mention that the LTx patients could not be examined in a dental office, in order to provide accurate assessments for oral health. Despite all limitations, these results provide valuable information for dental and medical professionals in terms of oral and systemic complications prevention, based on salivary monitoring, in liver transplant recipients.

## 5. Conclusions

The similar findings regarding concentrations of salivary proteins, as well as the proteolytic activity and salivary secretion rates of the two groups evaluated, suggest that salivary glands function was not affected in liver transplant recipients. In addition, the results obtained from the self-reported questionnaire suggest comparable oral health features in both groups. Oral health care is an important issue to be addressed after liver transplantation, considering the high risk for developing systemic complications. Further longitudinal studies including assessments of specific salivary proteins and clinical periodontal assessments are needed for a better understanding of the influence of the acid-base balance and the long-term impact of the immunosuppressive treatment on the salivary biochemistry and oral health after liver transplantation.

## Figures and Tables

**Table 1:** Background of the study groups.

Variables	LTx Patients	Healthy Controls
Age Mean (SD)	54.4 (9.4)	53.3 (9.7)
Males N (%)	11 (42.3)	7 (26.9)
Smoking N (%)	3 (11.5)	5 (19.2)
Rural N (%)	5 (19.2)	1 (3.8)
Tertiary education N (%)	18 (69.23)	17 (65.38)
Frequency of dental check-ups per year		
N (%)		
0	14 (53.85)	10 (38.46)
1	3 (11.54)	6 (23.08)
2	9 (34.62)	10 (38.46)

SD, standard deviation; LTx, post-liver transplant.

**Table 2:** Frequency distributions of responses to the 8 items of the Self-Reported Oral Health (SROH) questionnaire among liver transplant (LTx) patients and healthy controls.

SROH QUESTION (Abbreviation)	LTx Patients	Healthy Controls	*p*-Value
**Q1. Have gum disease**			
Yes	6 (50%)	6 (50%)	0.78 ^1^
No	13 (46.43%)	15 (53.57%)	
Don’t know	7 (58.33%)	5 (41.67%)	
**Q2. Teeth/gum health**			
Poor	6 (54.55%)	5 (45.45%)	0.88 ^2^
Fair	4 (36.36%)	7 (63.64%)	
Good	9 (47.37%)	10 (52.63%)	
Very good	4 (57.14%)	3 (42.86%)	
Excellent	1 (100%)	0	
Don’t know	2 (66.67%)	1 (33.33%)	
**Q3. Had gum treatment**			
Yes	11 (44%)	14 (56%)	0.41 ^1^
No	15 (55.56%)	12 (44.44%)	
**Q4. Loose tooth**			
Yes	9 (52.94%)	8 (47.06%)	0.17 ^2^
No	14 (43.75%)	18 (56.25%)	
Don’t know	3 (100%)	0	
**Q5. Lost bone**			
Yes	3 (33.33%)	6 (66.67%)	0.29 ^2^
No	23 (54.76%)	19 (45.24%)	
Don’t know	0	1 (100%)	
**Q6. Tooth does not look right**			
Yes	4 (44.44%)	5 (55.56%)	0.73 ^2^
No	22 (52.38%)	20 (47.62%)	
Don’t know	0	1 (100%)	
**Q7. Floss use**			
Yes	7 (31.82%)	15 (68.18%)	0.02 ^1,^*
No	19 (63.33%)	11 (36.67%)	
**Q8. Mouthwash use**			
Yes	12 (34.29%)	23 (65.71%)	0.003 ^2,^*
No	14 (82.35%)	3 (17.65%)	

^1^ Pearson Chi-squared test; ^2^ Fisher exact test; * statistical significance.

## Data Availability

The data presented in this study are available from the corresponding authors upon reasonable request.
